# Severe Hyperkalemia, a Case Report

**DOI:** 10.21980/J8KH1D

**Published:** 2020-07-15

**Authors:** Daniel Johnson, Dan Wiener

**Affiliations:** * Morristown Medical Center, Department of Emergency Medicine, Morristown, NJ

## Abstract

This case highlights the classic electrocardiogram (ECG) finding of a severely widened QRS complex with a sinusoidal pattern indicative of severe hyperkalemia. It also emphasizes the importance of the ECG in screening for electrolyte abnormalities and the ability to begin therapy before laboratory confirmation in the correct clinical setting. A 78-year-old male with history of end stage renal disease presented with chest pain. Findings on initial ECG allowed for rapid diagnosis and treatment before serum potassium levels were confirmed. His treatment consisted of cardiac stabilization with calcium, followed by efforts to shift potassium intracellularly with insulin and beta-agonists. Ultimately the patient was confirmed to have a severely elevated potassium level of eight mmol/L. Unfortunately, during aggressive initial management, the patient suffered a pulseless electrical activity cardiac arrest, and in accordance with his wishes no resuscitative efforts were performed. While the outcome was unfortunate, it also highlights the temporal relationship between the presented ECG findings and fatal arrhythmias. After reviewing the case, one should recognize the importance of the ECG for screening and prompt treatment of electrolyte derangements, understand the acute management of hyperkalemia, and appreciate the possibility for rapid deterioration.

**Topics:**

Hyperkalemia, electrocardiography, electrolytes.

**Figure f1-jetem-5-3-v1:**
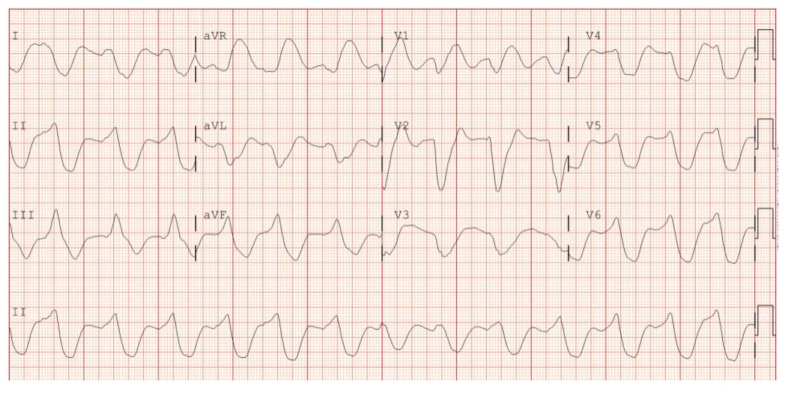


## Introduction

[Fig f1-jetem-5-3-v1]Hyperkalemia is a life-threatening electrolyte abnormality associated with the increasing prevalence of chronic renal failure and medical management of hypertension and cardiovascular disease.^1^ It has been estimated that 3.7 million patients per year are diagnosed with evident laboratory hyperkalemia.^2^ Frequently in the emergency department, the initial ECG is abnormal, and identification of subtle variations could avoid treatment delays associated with awaiting laboratory values.^1^ This case outlines the early diagnosis of severe hyperkalemia with the rarely encountered but nearly pathognomonic electrocardiographic finding of a severely widened QRS complex with “Sine Wave” morphology.

## Presenting concerns and clinical findings

This is a 78-year-old male with a history of end-stage renal disease on hemodialysis who presents to the ED complaining of chest discomfort refractory to home doses of sublingual nitroglycerine. He has been receiving regular hemodialysis and is due for dialysis on the day of the presentation. Despite close attention to his renal disease, he has developed several episodes of hyperkalemia in the past requiring treatment. In addition to his renal disease, he has severe coronary artery disease for which he is not a candidate for percutaneous or surgical revascularization. He presented to the emergency department due to severe chest discomfort limiting his ability to perform his normal activities and not responding to home medications.

## Significant findings

The initial ECG obtained upon arrival shows what is commonly referred to as a sine wave pattern. This patient does have a biventricular pacemaker which would ordinarily create a wide QRS complex mimicking an intraventricular conduction delay. However, the QRS complex here is exceptionally wide, in excess of 400 milliseconds (normal: less than 120 milliseconds). As the QRS widens, alongside other deflections present on the ECG, it morphologically mimics a mathematical sine wave.

## Patient course

The patient’s clinical history and concerning ECG findings quickly prompted empiric treatment of presumed hyperkalemia. Simultaneously, point of care testing was ordered, while one gram of calcium chloride was administered via IV push (IVP). The initial point of care potassium was found to be 7.8 mmol/L. Ten units of regular insulin with 25 grams of 50% dextrose were administered via IVP, while ten mg of albuterol was nebulized. Nephrology was consulted for emergent hemodialysis. After cardiac stabilization with an initial dose of calcium, the QRS duration was noted to narrow slightly. However, the patient subsequently developed a pulseless electrical activity cardiac arrest, and his family requested that the patient not be resuscitated in accordance with his wishes. It was later confirmed on initial chemistries drawn that the patient serum potassium was eight mmol/L.

## Discussion

This case report highlights the critical nature of recognizing signs of hyperkalemia on ECG and prompts a conversation regarding the correlation of ECG findings with serum potassium levels. Often, an ECG is the first screening test and the most rapidly available information to screen for hyperkalemia while awaiting laboratory testing.^3^ As serum potassium levels rise, repolarization abnormalities and sinoatrial node dysfunction result in predictable alterations to the ECG tracing. Initially, peaking of the T waves is followed by flattening of the P wave. This then degenerates into prolongation of the PR segment, cessation of SA node activity altogether, QRS widening, and finally fatal ventricular arrhythmias.^4^,^5^ We can attempt to correlate these specific findings with potassium levels; however, this is challenging and, as some studies suggest, unreliable. Cohen et al. highlighted that even “classic” ECG findings can be absent through every range from mild to severe hyperkalemia. In their analysis, no particular ECG finding could be attributed to a specific serum potassium level with any statistical significance.^6^ The ECG pattern described above can be confounded by underlying conduction abnormalities, electrolyte abnormalities, and medications that impact normal electrical conduction.^2^

To briefly discuss the emergency management of hyperkalemia, the foundation of therapy revolves around immediate cardiac membrane stabilization followed by shifting of potassium intracellularly and finally eliminating excess potassium from the body. Calcium initially functions to stabilize the membrane of cardiac myocytes and prevent arrhythmia. While calcium chloride can be sclerosing to veins and potentially dangerous in the event of infiltration, it contains more significant amounts of elemental calcium. In general, calcium gluconate is considered to be safer when administered peripherally; however, one study performed in the setting of a calcium gluconate shortage showed a minimal number of complications when calcium chloride was administered peripherally.^7^ Shortly after that, potassium can be shifted intracellularly by the function of insulin on cellular membrane channels and beta agonism, typically with nebulized albuterol.^8^ The use of potassium losing diuretics, cation exchange resins, and sodium bicarbonate are common adjunctive treatments for hyperkalemia. Diuresis and cation exchange are methods that, similar to hemodialysis, are able to decrease total body potassium. Sodium polystyrene sulfonate is a common cation exchange resign that facilitates potassium removal from the gastrointestinal tract when administered orally or rectally. Loop diuretics such as furosemide facilitate renal excretion of potassium in the ascending Loop of Henle. Similar to other acute interventions, sodium bicarbonate promotes intracellular shift of potassium through an increase in blood pH. The utility of these modalities in the acute stabilization of patients with hyperkalemia is controversial. Their ability to quickly, reliably, and safely lower plasma potassium levels is not consistently demonstrated in the literature.^9^ While these adjunctive therapies are often discussed and utilized in conjunction with consultants, primary management should be focused on the immediate cardiac membrane stabilization and intracellular shift of potassium as described above. Ultimately, shifting potassium to the intracellular compartment is temporary, and definitive treatment involves elimination with hemodialysis.^10^

In summary, this case outlines the utility of the initial ECG in the screening for hyperkalemia. While it is generally agreed upon that the ECG is unreliable at predicting a serum potassium level, classic abnormalities may be the only indication of severe derangements while awaiting the results of serum testing. Lifesaving treatment can and should begin promptly in the correct clinical setting before confirmation of serum levels.

## Supplementary Information


