# Supplementary figures and images for: Severe Hyperkalemia, a Case Report
Source: J Educ Teach Emerg Med. 2020 Jul 15;5(3):V1–3. doi: 10.21980/J8KH1D (PMC10332545; doi:10.21980/J8KH1D)

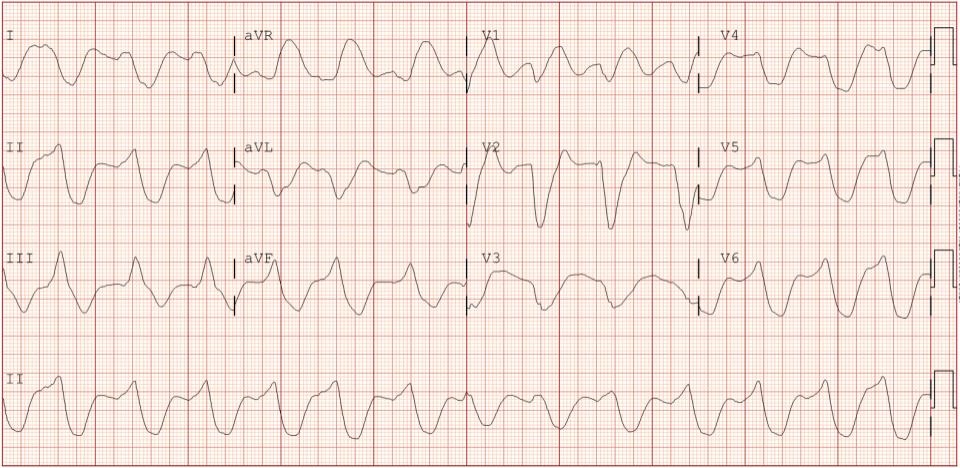

Supplement: Supplementary file 1 [file jetem-5-3-v1-supp1.jpg]
